# Metabolomic signatures and microbial community profiling of depressive rat model induced by adrenocorticotrophic hormone

**DOI:** 10.1186/s12967-019-1970-8

**Published:** 2019-07-15

**Authors:** Jing Song, Weini Ma, Xinyi Gu, Le Zhao, Jiaye Jiang, Ying Xu, Lei Zhang, Mingmei Zhou, Li Yang

**Affiliations:** 1 Center for Chinese Medicine Therapy and Systems Biology, Institute for Interdisciplinary Medicine Sciences, Shanghai University of Traditional Chinese Medicine, 1200 Cailun Road, Pudong District, Shanghai, 201203 China; 2 Experiment Center of Teaching & Learning, Shanghai University of Traditional Chinese Medicine, Shanghai, 201203 China; 3 School of Pharmacy, Shanghai University of Traditional Chinese Medicine, Shanghai, 201203 China; 4 Department of Physiology, Shanghai University of Traditional Chinese Medicine, Shanghai, 201203 China; 5 Shanghai Innovation Center of TCM Health Service, Shanghai University of Traditional Chinese Medicine, Shanghai, 201203 China

**Keywords:** Depression, HPA axis, Gut microbiota, Metabolomics

## Abstract

**Background:**

Adrenocorticotrophic hormone (ACTH)-treatment rat model has been utilized as a widely accepted model of treatment-resistant depression. Metabolomic signatures represent the pathophysiological phenotype of diseases. Recent studies in gut microbiota and metabolomics analysis revealed the dramatic role of microbiome in psychoneurological system diseases, but still, the mechanisms underlying gut microbiome–host interaction remain unclear.

**Methods:**

Male Wistar rats were *s.c*. injection of ACTH fragment 1–24 for 14 days to induce treatment-resistant depression. Depression-related behavioral tests, analysis of serum monoamine neurotransmitters and hypothalamic–pituitary–adrenal (HPA) axis-related hormones were determined for assessment of ACTH-induced depression rat model. A gas chromatography-time-of-flight mass spectrometer based urinary metabolomic signatures integrated 16S rRNA sequence analysis based gut microbial profiling was performed, as well as Spearman’s correlation coefficient analysis was used to manifest the covariation between the differential urinary metabolites and gut microbiota of genus level.

**Results:**

Chronic injection of ACTH-induced depression-like phenotype (increased immobility time in forced swimming test and tail suspension test) was accompanied by peripheral serotonin down-regulation and HPA axis overactivation (ACTH and corticosterone up-regulation). Urinary metabolomics analysis indicated that pyruvic acid, l-threonine, mannitol, d-gluconic acid, 4-hydroxybenzoic acid, d-arabitol, myo-inositol and ascorbic acid levels were reduced in ACTH-treated rats’ urine, while hippurate level was elevated. In addition, microbial community profiling revealed bacterial enrichment (e.g. *Ruminococcus*, *Klebsiella*) and reduction (e.g. *Akkermansia*, *Lactobacillus*) in the ACTH-induced depression rat model. Correlation analysis showed that *Akkermansia* and *Lactobacillus* were closely relevant to metabolites myo-inositol and hippurate, which were included in host inositol phosphate metabolism, and phenylalanine, tyrosine and tryptophan biosynthesis.

**Conclusions:**

Depression rat model induced by ACTH is associated with disturbance of pyruvate metabolism, ascorbate and aldarate metabolism, inositol phosphate metabolism, glycine, serine and threonine metabolism, and glycolysis or gluconeogenesis, as well as changes in microbial community structure. Gut microbiota may participate in the mediation of systemic metabolomic changes in ACTH-induced depression model. Therefore, integrated metabolomic signatures and gut microbial community profiling would provide a basis for further studies on the pathogenesis of depression.

**Electronic supplementary material:**

The online version of this article (10.1186/s12967-019-1970-8) contains supplementary material, which is available to authorized users.

## Background

As the latest World Health Organization (WHO) report showed, depression is a high-risk psychiatric disease that affects people of all races around the world, and ranks among the world’s largest contributors of years lived with disability. It is also one of the major causes of the global economic burden of disease [1]. However, up to 60% of patients show no respond to the initial antidepressant treatment, which is called treatment resistant depression (TRD), which may be a subtype of depression patients with unique pathophysiological features [2, 3]. Due to lack of sufficient relief from current antidepressant therapy, TRD patients have an increasing risk of suffering from chronic psychosocial disorders, relapses and suicide [4].

The abnormality of hypothalamic–pituitary–adrenal (HPA) axis is one of the most concerned research areas in depression [5–7]. Stress can activate the HPA axis, including increased release of corticotropin-releasing hormone (CRH) and adrenocorticotropic hormone (ACTH) [8, 9]. In clinical studies, the dysregulation of HPA axis is also a common feature found in depressive patients [10]. Numerous CRH receptors are resident in extra-hypothalamic area, and most cells of the 5-hydroxytryptamine (5-HT) and noradrenaline (NA) systems are also included herein [11]. CRH can activate 5-HT neurons in the dorsal rape nucleus, and regulate NA activity. Glucocorticoids can stimulate 5-HT and NA systems via mineralocorticoid and glucocorticoid receptors. Therefore, the disorder of HPA axis and its interaction with central monoaminergic system might be one of the main causes of TRD [12, 13].

As an interesting animal model of TRD, chronic administration of ACTH to rodent, mimicking the stress induced the HPA axis activation, results in resistance to the treatment of imipramine in the forced swimming test (FST) [14], as well as resistance to other antidepressants [15, 16]. Though, ACTH-induced depression rat model has been widely accepted as an animal model for TRD, the underlying mechanism of this model is not completely understood.

Deregulation of the gut microbial community has been extensively associated with the diseases of the central nervous system (CNS). The crosstalk between the gut microbiota and the CNS, so-called gut microbiota–brain axis, occurs through neuroendocrine, enteric, autonomic, and immune system pathways, and may impact the host physiological and pathological mechanisms, including the HPA axis activation, and the communications between neurotransmitters and immune system [17]. Therefore, the perturbed gut microbiota could be associated with neurological diseases via the gut microbiota–brain axis [18]. The alterations of specific species of gut microbiota may contribute to the occurrence and the development of depression, while depressive states may cause the changes of specific species of gut microbiota, which eventually result in more severe depression. These specific species of gut microbiota are mainly included in the bacteria phyla of *Firmicutes*, *Actinobacteria*, *Bacteroidetes* and *Proteobacteria* [19]. Meanwhile, a number of studies have shown that probiotics, such as *Lactobacillus* and *Bifidobacterium*, have beneficial psychoneurological effects [20, 21]. It is reported that CBM588, a specific phenotype of the strain *Clostridium butyricum*, in combination with antidepressants is effective and well tolerated in the treatment of TRD [4]. Recently, a 16S ribosomal RNA gene sequencing of feces based study showed that the antidepressant effects of ketamine, an *N*-methyl-d-aspartate receptor (NMDAR) antagonist showed rapid and sustained antidepressant effects in TRD patients, might be partly mediated by the restoration of altered gut microbiota [22].

The crosstalk between gut microbiota and brain might be indirect. The metabolites of gut microbiota, which circulate in the systematic circulation and metabolism, may play a vital role in the gut microbiota–brain axis. Metabolomics is a systematic biological method for measuring the changes of endogenous small molecule metabolites. It has been increasingly applied to the diagnosis of diseases, the discovery of disease biomarkers, and the pathogenesis study of diseases [23]. In metabolomics studies, urinary sample is non-invasive, and more convenient for collection compared to other samples (blood or specific organ). Furthermore, the greatest advantage of urinary sample for metabolomics studies is that it contains a combination of metabolites in a relatively longer window period (12 or 24 h), while the metabolites in blood can only represent the transient state of the research object. At present, urinary samples of animal or human have been widely used for metabolomics research to reveal the pathophysiological mechanism of diseases systemically [24]. Therefore, to characterize the global characteristics of ACTH-induced TRD, it is necessary to combine the gut microbiota study with the metabolomic signatures.

In the present study, an integrative gas chromatography-time-of-flight mass spectrometer (GC-TOFMS)-based urinary metabolomics and 16S rRNA gene sequencing approach was performed on chronic ACTH-treated rat model with depression phenotype to reveal the differentiated gut microbiota and urinary metabolites. Further, the relevancies of above two were discovered to reveal the interactions between the gut microbiota and the host metabolism in ACTH-induced TRD rats. The results will help to enhance comprehensive understanding of the pathogenesis of ACTH-induced TRD.

## Materials and methods

### Animals and treatments

A total of 20 male Wistar rats (10 weeks, 230–250 g) were obtained from Shanghai Sippr-BK Laboratory Animal Co. Ltd., China. The animals were under a constant light–dark cycle (07:00–19:00 at 40 W light condition), with free access to food and water ad libitum in a standard laboratory environment (20 ± 5 °C and 55 ± 15% humidity). All the study was conducted under the Guidelines for Animal Experimentation of Shanghai University of Traditional Chinese Medicine.

The rats were randomly divided into two groups: normal control group (Control, n = 10) and ACTH-treated TRD model group (ACTH, n = 10). ACTH group received *s.c*. injection of adrenocorticotropic hormone fragment 1–24 (100 μg/rat, 14 days, CAS No. 16960-16-0, purchased from Sigma Chemical Co., MO, USA) [25], and at the same time, Control group received the same volume of saline injection.

### Behavioral test

#### Forced swimming test

Forced swimming test (FST) [26], a representative behavioral test for depression, is frequently used to evaluate the depressive state in rodent models. Forced immersion of rats in water for an extended period produces a characteristic behavior of immobility, and rats of depression are more inclined to exhibit a longer immobility time in a test period. After 14 days of ACTH-treatment, each rat was placed in a transparent glass container (50 cm height × 18 cm diameter) filled to 30 cm with 25 ± 1 °C water. Rats were forced to swim for 6 min and the duration of immobility within the last 4 min was measured. All behavioral tests were performed between 14:00 and 18:00 p.m. Immobility was defined as floating in the water without struggling and only keeping the head above the water without other motions. The immobility time was determined by an observer blinded to the purpose of the experiment and the water must be changed after every test.

#### Tail suspension test

Tail suspension test (TST) [27] is based on the fact that animals subjected to the short-term, inescapable stress of being suspended by their tail, will develop an immobile posture. Each rat was suspended 50 cm above the floor by tape. Every test was performed for 6 min and the duration of immobility within the last 4 min was measured. Immobility means that the animal gives up its active struggle and has not moved at all. Immobility time was determined by an observer who was unaware the purpose of the test.

### Sample collection and preparation

After the last behavior test, all rats were fasted and placed in metabolic cages individually 24 h for urinary sample collection. The urinary samples were centrifuged at 1500×*g* for 5 min at 4 °C. The resultant supernatants were stored at − 80 °C pending GC-TOFMS analysis. As for fecal samples, approximately 2 pellets of feces were attained from each rat, then transferred into EP tubes, and stored at − 80 °C until further analysis. Blood was collected from the abdominal aorta after anesthesia by chloral hydrate (300 mg/kg body weight, *i.p*.). Blood samples were centrifuged at 3000×*g* for 15 min at 4 °C, and the supernatants were stored at − 80 °C.

### Serum parameters analysis

Serum samples were conducted for monoamine neurotransmitter and hormone analysis with ELISA kits, such as 5-HT, norepinephrine (NE), CRH, ACTH, and corticosterone (CORT), according to the manufacturer’s protocols (Cusabio Biotechnology Co., Ltd, Wuhan, China).

### Urinary metabolomic signatures

According to our previous study [28], metabolomics analysis of urinary samples was based on a GC-TOFMS metabolomics method. Rat urinary samples were thawed at room temperature 2 h before the experiment and 2.0 mL urinary samples were taken in an EP tube and centrifuged at 12,000 rpm for 10 min. 100 μL of supernatant was subjected to urea degradation by adding urease (70 IU, 37 °C, 15 min), and then pretreated with l-2-chlorophenyl alanine as an internal standard and ethyl chloroformate as a derivatizing agent. The sample analysis was performed using GC-TOFMS (Pegasus HT, Leco Corp., St. Joseph, MI; electron ionization mode).

### Microbial community profiling

To analyze the taxonomic composition of the microbial community, 16S rRNA sequencing of fecal sample was performed following the method we published previously with minor modifications [29]. Total DNA in fecal samples was extracted using E.Z.N.A.^®^ soil DNA Kit (Omega Bio-Tek, Norcross, GA, USA). The quality of DNA extraction was detected by 0.8% agarose gel electrophoresis and DNA was quantified using an ultraviolet spectrophotometer. The V3–V4 region of the bacteria 16S rRNA gene was amplified by PCR using Q5 High-Fidelity DNA polymerase (NEB, USA) and primers 520 F (5′-AYTGGGYDTAAAGNG-3′) and 802 R (5′-TACNVGGGTATCTAATCC-3′). The amplification products of PCR were qualified by 2% agarose gel electrophoresis, and purified by AxyPrep DNA Gel Extraction Kit (AXYGEN Biosciences, Union City, CA, USA). Then the amplification products were quantified on Microplate reader (BioTek, FLx800) using Quant-iT PicoGreen dsDNA Assay Kit (Invitrogen, USA). The purified amplicons were finally combined at equimolar concentrations and paired-end sequencing was performed on an Illumina MiSeq platform (Majorbio Bio-Pharm Technology Co. Ltd., Shanghai, China).

### Statistical analysis

The raw data from GC-TOFMS analysis were exported in NetCDF format using the Chroma TOF software (v4.44, Leco Co., Los Angeles, CA), and further preprocessed by R 2.13.2 (Lucent Technologies). Then the peak area, retention time and compounds name were included in output data for multivariate analysis using SIMCA-P 11 software (Umetrics, Umea, Sweden) to perform principal component analysis (PCA) and orthogonal partial least squares discriminant analysis (OPLS-DA). Furthermore, OPLS-DA was used to select the differential metabolites with variable importance in the projection (VIP) value > 1.0. The significance identification was then performed by Student’s t test (*P* < 0.05) and fold change (FC, compared to Control group, a range of > 1.2 or < 0.8). Kyoto Encyclopedia of Genes and Genomes (KEGG, http://www.genome.jp/kegg/) and Human Metabolome Database (HMDB; http://www.hmdb.ca/) were applied to confirmed potential depression biomarkers. The metabolic pathway analysis module of MetaboAnalyst 3.0 (http://www.metaboanalyst.ca/) was used to identify the metabolic pathways of differential metabolites.

For sequencing data, Trimmomatic, a flexible read trimming tool for Illumina sequence data, was used to quality-filter the raw fastq files. The reads were truncated in a sliding window of 10 bp with an average quality score > 20. The paired-end sequences passed through the quality-filter were merged using FLASH (v1.2.7, http://ccb.jhu.edu/software/FLASH/). Bases mismatch was not allowed. The sequence of each sample was isolated based on the barcode (exactly matching) and the primer (exactly matching); readings containing ambiguous bases were discarded. Operational Taxonomic Units (OTUs) were clustered with 97% similarity cut-off using UPARSE (v7.1 http://drive5.com/uparse/). The taxonomy of each 16S rRNA gene sequence was classified against the Greengenes database using a confidence threshold of 70%. Partial least squares discriminant analysis (PLS-DA) was used to find out whether ACTH-treated rats could be separated from the control rats. Furtherly, the linear discriminant analysis (LDA) effect size (LEfSe) method was used to identify the dominant phylotypes responsible for differences between ACTH group and Control group. LEfSe aims to provide biomarkers that explain the effects of biomarker discovery comparisons and hypothesis-driven researches on phenotypes of interest (two or more). The visualization of the biomarkers found on taxonomic trees provides an effective tool of concluding the results in a biologically meaningful manner, because it captures statistically and visually the hierarchical relationships inherent in 16S-based taxonomies/phylogenies or in pathways and biomolecular functions.

The data was analyzed using SPSS 21.0. Statistical significance among the groups was conducted by two-tailed Student’s t-test. All values were expressed as the mean ± standard deviation (SD). Probability values of less than 0.05 were considered to show a statistical significance.

### Correlation analysis between metabolomic signatures and microbial community profiling

To explore the relationship between altered gut microbiota and perturbed urinary metabolites, correlation coefficient of Spearman’s between metabolomic signatures and gut microbial alterations of genus level were exhibited in the form of a heat map. Coefficient r values were distinguished by different color blocks. Additionally, the metabolic associations of each well-correlated members of gut microbe (|*r*| > 0.3) were visualized as a cross-correlation diagram showing positive (in red line) and negative (in blue line) connections.

## Results

### Quality assessment of ACTH-induced depression model

Combined FST and TST results were used to access the depressive state of ACTH model. As shown in Additional file 1: Fig. S1, compared to Control group, immobility time of rats treated with ACTH in FST and TST was significantly increased (*P* < 0.01).

NE and 5-HT are monoamine neurotransmitters closely associated with depression. Serum 5-HT and NE levels of two groups were shown in Additional file 1: Fig. S2. As the results of *s.c.* injection of ACTH, serum concentration of 5-HT was significantly decreased (*P* < 0.01) in ACTH group, compared with Control group. At the same time, the level of NE in ACTH group showed a slight decrease trend with no significance.

CRH, ACTH and CORT are the major hormones in the HPA axis. In our study, ACTH and CORT levels were markedly increased in the serum of ACTH-treated rats compared to Control group (*P* < 0.001). The CRH level of ACTH group was lower than that in Control group, however there was no statistical difference (*P* > 0.05) (Additional file 1: Fig. S2). This may be the result of negative feedback from ACTH injection.

### Urinary metabolomic signatures of ACTH-treated rats

**Fig. 1:**
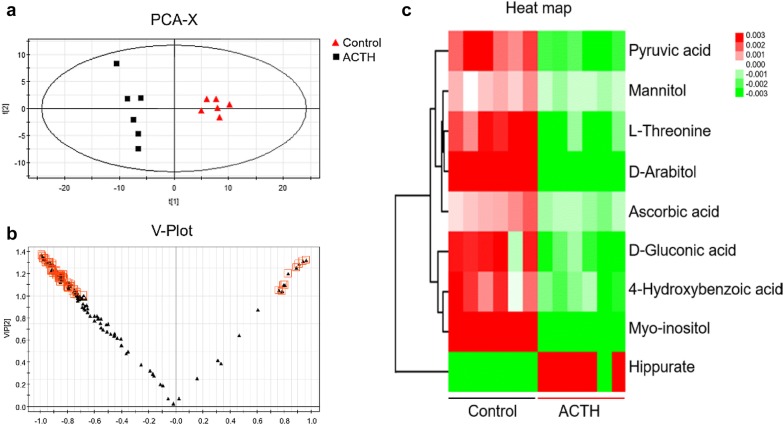
Urinary metabolic profiling (n = 6). PCA score plot (**a**), V-plots (**b**, VIP > 1.0), and heat map (**c**) of the differential metabolites in ACTH group and Control group

Urinary metabolomics is a typical metabonomic research approach, which is characterized by convenient collection, non-injury, and continuity of metabolites. As shown in Fig. 1a, the metabolomic signatures of ACTH-treated rats were remarkably separated from Control group in PCA (*R*^2^X = 0.540, *Q*^2^ = 0.43) and OPLS-DA score plots (*R*^2^X = 0.646, *R*^2^Y = 0.988, *Q*^2^ = 0.955, Additional file 1: Fig. S3), suggesting that ACTH injection caused significant metabolic changes. In addition, the variables with VIP > 1 (surrounded by red boxes) in V-plot (Fig. 1b) were further analyzed. We finally identified 9 metabolites. Compared with Control group, the levels of pyruvic acid, l-threonine, mannitol, d-gluconic acid, 4-hydroxybenzoic acid, d-arabitol, myo-inositol, ascorbic acid were lower in ACTH group, and the level of hippurate was increased (Fig. 1c, Additional file 2: Table S1).

### Metabolic pathway and network analysis

**Fig. 2:**
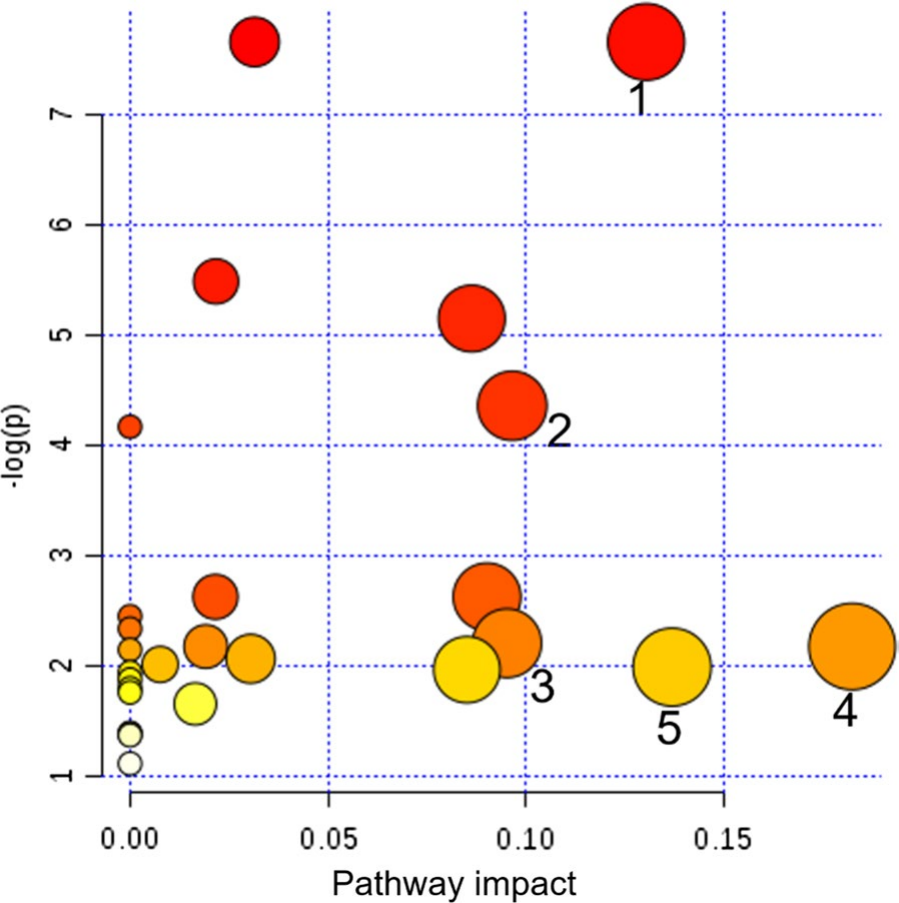
Pathway analysis of urinary metabolites using Metaboanalyst (impact factor ≥ 0.1). (1) Ascorbate and aldarate metabolism; (2) glycine, serine and threonine metabolism; (3) glycolysis or gluconeogenesis; (4) pyruvate metabolism; (5) inositol phosphate metabolism

**Fig. 3:**
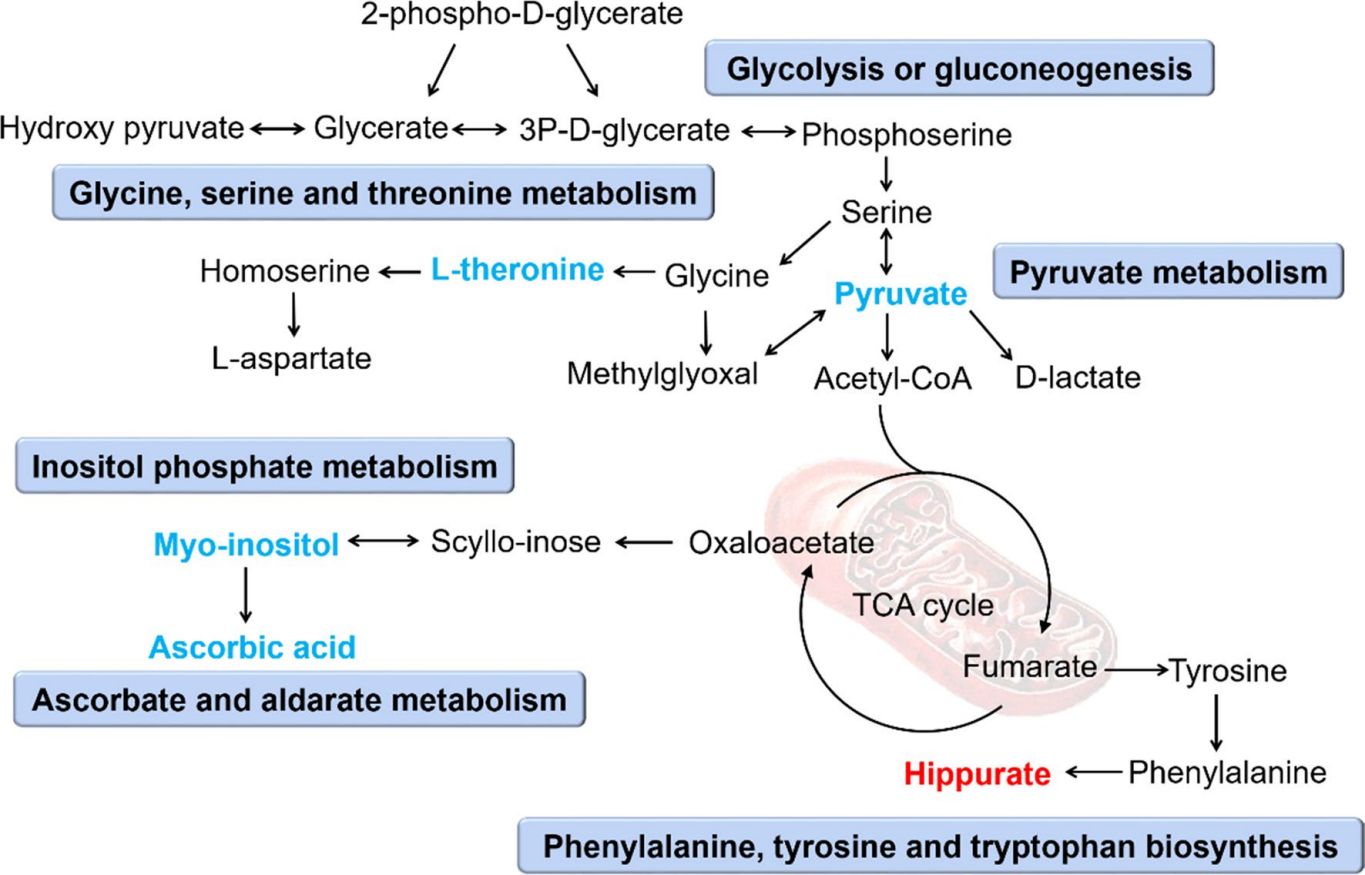
Perturbed metabolic pathways and altered urinary metabolites in ACTH-treated rats. Red labeled metabolites were up-regulated, and blue labeled metabolites were down-regulated. Blue blocks represented disturbed metabolic pathways

KEGG and HMDB were used to link the 9 urinary differential metabolites to potential relevant pathway, and MetaboAnalyst 3.0 was applied to identify the impact value of relevant pathways. The results showed that five main metabolic pathways were affected, including pyruvate metabolism, ascorbate and aldarate metabolism, inositol phosphate metabolism, glycine, serine and threonine metabolism, and glycolysis or gluconeogenesis (Fig. 2, impact factor ≥ 0.1). Based on the relationship between metabolomic signatures, the disturbed metabolic pathways were represented in diagram (Fig. 3).

### Microbial community profiling of ACTH-induced depression

**Fig. 4:**
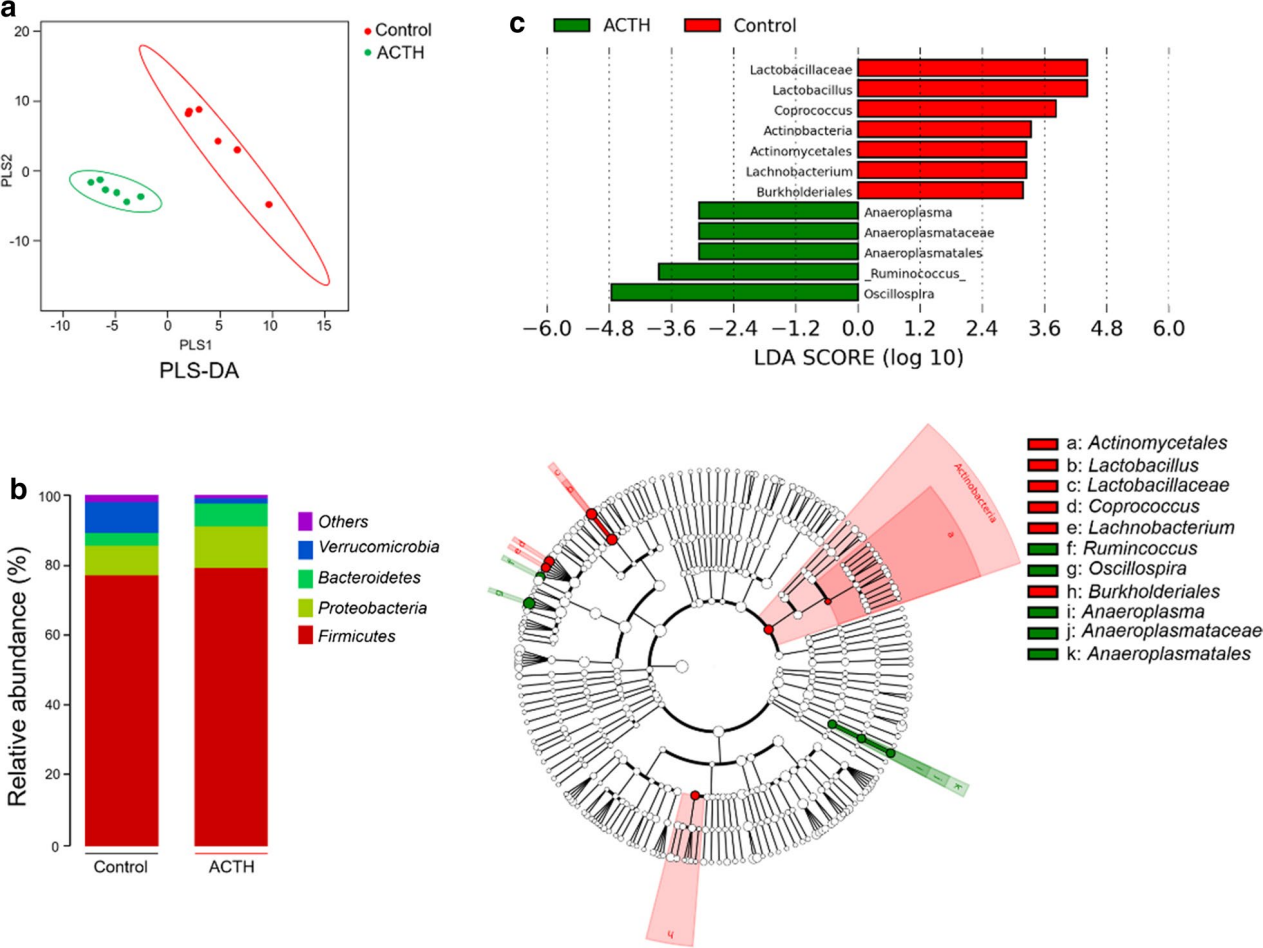
**a** The partial least squares discriminant analysis (PLS-DA) of the microbial community for ACTH group (in green) and Control group (in red), n = 6. **b** The fecal microbial structures at the phylum level for ACTH group and Control group, different colors represent different microbe at phylum level, n = 6. **c** LEfSe analysis of gut microbiota for ACTH group and Control group, n = 6. The abundances of taxa from phylum to genus levels were compared between the two groups. Taxa enriched in ACTH group are indicated by a negative LDA score (green), and taxa enriched in Control group have a positive LDA score (red). Only taxa of an LDA significant threshold of 3.0 were shown

In order to analyze whether there was a difference in the structure of the microbial community in the gut microbiota between ACTH group and Control group, PLS-DA analysis was performed as shown in Fig. 4a. The results showed ACTH group had a distinct microbiota composition that clustered differently from Control groups. Moreover, the microbial community structures at the phylum level of ACTH group and Control group were shown in Fig. 4b. The abundance of each sample is presented as the percentage of the total number of sequences. In ACTH-treated rats, the relative abundances of *Firmicutes* (*P* < 0.05) and *Bacteroidetes* (*P* < 0.01) were increased significantly, and the relative abundance of *Verrucomicrobia* (*P* < 0.01) was decreased significantly. In addition, the ratio of *Firmicutes*-to-*Bacteroidetes* in ACTH group was higher than that of Control group (Additional file 1: Fig. S4). At the genus level (Additional file 1: Fig. S5), gut microbiota of two groups were both dominated by *Ruminococcus*, *Klebsiella*, *Lactobacillus*, and *Akkermansia*. The relative abundances of *Ruminococcus* (*P* < 0.05) and *Klebsiella* (*P* < 0.01) in ACTH group were significantly higher than those in Control group, but the relative abundances of *Lactobacillus* (*P* < 0.01) and *Akkermansia* (*P* < 0.01) were decreased significantly. The metagenome analysis of LEfSe approach was applied to explore the differential gut microbiota (from phylum to genus) in two groups of rats (Fig. 4c, LDA score > 3.0). *Anaeroplasma*, *Anaeroplasmataceae*, *Anaeroplasmatales*, *Rumincoccus* and *Oscillospira* were the most abundant differential microbiota in ACTH group. In Control group, *Lactobacillus*, *Lactobacillaceae*, *Coprococcus*, *Actinobacteria*, *Actinomycetales*, *Lachnobacterium* and *Burkholderiales* were significantly increased compared to ACTH group.

### Correlation analysis between metabolomic signatures and microbial community profiling

**Fig. 5:**
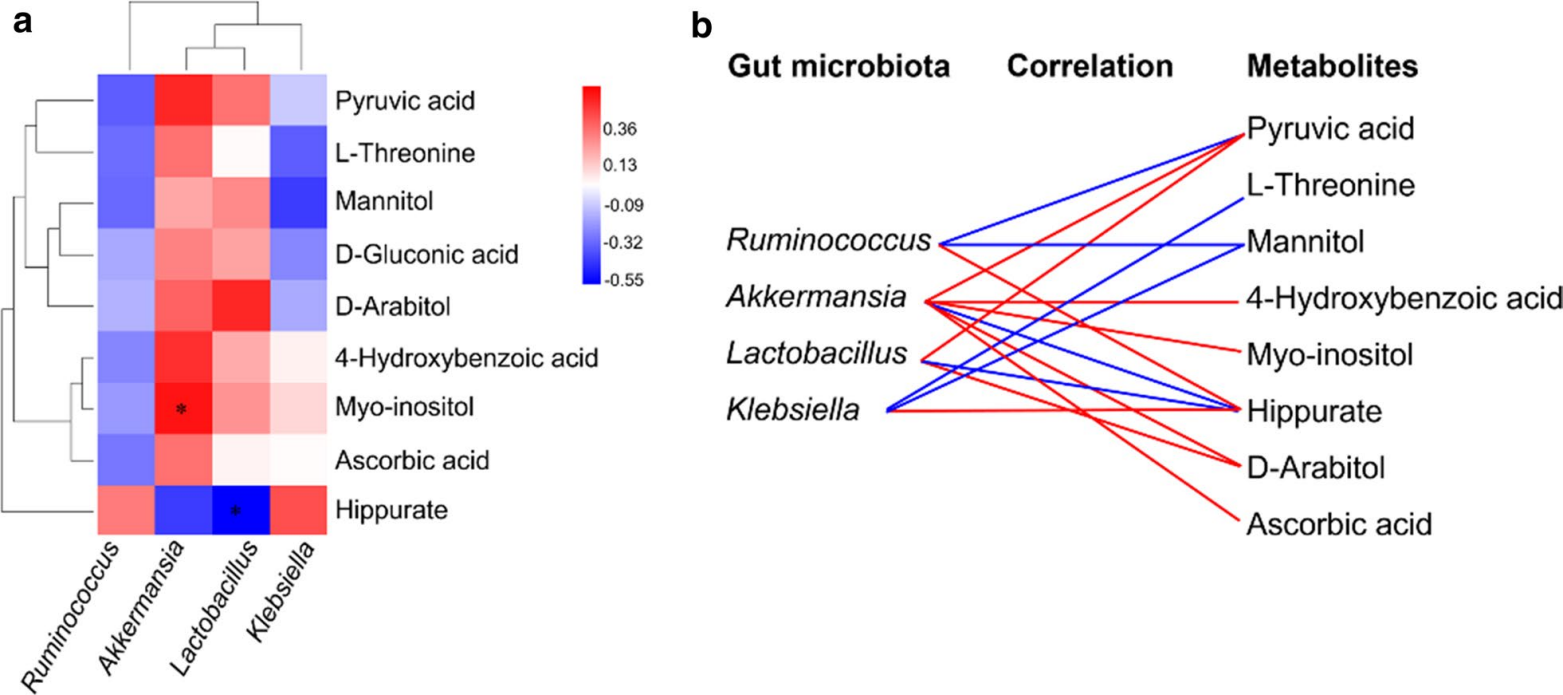
The relevance between the gut microbiota of genus level and the differential urinary metabolites. **a** Spearman’s correlation heat map: red indicates positive correlation, while blue indicates negative correlation. The deeper color means the greater correlation (*P < 0.05). **b** The gut microbiota of genus level, predicted by metabolic variation (|*r*| > 0.3), is labeled with a similarity value. Lines connecting with metabolites show the direction of relevance to each genus of microbe with the red (positive) or blue (negative) lines

The covariation of urinary differential metabolites and the gut microbiota of genus level was presented as a heat map diagram (Fig. 5a). The metabolic connections of well-predicted bacteria in gut microbiota were shown in Fig. 5b (|*r*| > 0.3). There were multiple correlations between gut microbiota of genus level and metabolites, such as negative correlation between *Ruminococcus* and pyruvic acid (*r* = − 0.338), mannitol (*r* = − 0.313), and positive correlation with hippurate (*r* = 0.313). *Akkermansia* has a positive correlation with pyruvic acid (*r* = 0.5), l-threonine (*r* = 0.335), 4-hydroxybenzoic acid (*r* = 0.484), myo-inositol (*r *= 0.637, *P* < 0.05), d-arabitol (*r* = 0.370) and ascorbic acid (*r* = 0.336), but negatively related to hippurate (*r* = − 0.415). *Lactobacillus* was negatively correlated with hippurate (*r* = − 0.601, *P* < 0.05), but positively correlated with pyruvic acid (*r* = 0.334) and d-arabitol (*r* = 0.695, *P* < 0.05). In contrast, *Klebsiella* was positively associated with hippurate (*r* = 0.411) and negatively correlated with l-threonine (*r* = − 0.340) and mannitol (*r* = − 0.412).

## Discussion

Depression is a multifactorial disease which is regulated by genetic and environmental factors. As a systematic disease, it is a complex phenotype associated abnormalities ranging from central nervous system to many peripheral systems [30–32]. As an important component of the neuroendocrine system, HPA axis is a complex collection of direct influences and feedback interactions, and controls the response to stress and regulates many body processes including digestion, the immune system, mood and emotion, sexuality, energy storage and expenditure [33, 34]. In humans, gut microbiota contains the largest number of bacteria and the greatest number of species compared to other areas of the body. It not only plays a barrier to pathogenic organisms, but also serves as an endocrine organ by providing short-chain fatty acids (SCFAs), vitamin, and xenobiotics to the host [35]. Although the vital role of HPA axis dysfunction or gut microbiota dysregulation in pathogenesis of TRD has been widely accept, the interactions between the structural features of gut microbiota and host metabolic phenotype in depression accompanied by HPA axis dysfunction have rarely been reported until now. In the present study, an integrative metabolomic signatures and microbial community profiling was employed on ACTH-induced depression rats compared with normal control rats.

For FST and TST (Additional file 1: Fig. S1), striking helpless and depression-like behaviors observed in ACTH group were consistent with other reports [25, 36]. Elevated levels of ACTH and CORT in serum and negative feedback of CRH level (Additional file 1: Fig. S2) confirmed that ACTH injection induced hyperactivity of HPA axis, which was reported to be closely related to depression [37, 38].

The metabonomic results indicated that the differential metabolites were mainly related to disorders of energy metabolism, amino acid metabolism, ascorbate and aldarate metabolism, and inositol phosphate metabolism (Fig. 3). The disorder of energy metabolism was mainly related to pyruvate metabolism and glycolysis or gluconeogenesis. Pyruvic acid is an important intermediate in the glucose metabolism and its carboxylate anion is called pyruvate. Pyruvate is the end product of glycolysis and can be converted to acetyl coenzyme A (acetyl-CoA) by oxidative decarboxylation, which further participates in tricarboxylic acid (TCA) cycle to generate ATP [39]. It was reported that perturbed energy metabolism is ubiquitous in animal models of depression [40, 41]. In ACTH group, pyruvic acid concentration was significantly reduced (Additional file 2: Table S1), interfering with glycolysis or gluconeogenesis pathway, which may further lead to TCA circulatory dysfunction and depressive symptoms. Threonine, the essential dietary amino acid, is the precursor to isoleucine. Since Threonine is mainly found in the central nervous system, it can be of great help in the treatment of different types of depression. Threonine contributes to the synthesis of glycine and serine, and plays a key role in maintaining the integrity and barrier function of the intestinal mucosal [42]. Previous studies showed that abnormal amino acid metabolism might usually happen in depression and disturbed amino acid level might be served as clinical trait-biomarker for depression [43, 44]. Our research found that l-threonine level was significantly reduced in ACTH group and played a key role in the glycine, serine and threonine metabolic pathways, which may have great impact in the occurrence of depression (Additional file 2: Table S1). As the major metabolite of human, hippurate is formed by the reaction of many aromatic compounds such as benzoic acid and toluene with glycine and is involved in phenylalanine, tyrosine and tryptophan biosynthesis [45] (Fig. 3). Glycine is one of the excitatory amino acids closely related to major depression [46]. The increase of hippurate concentration is closely related to the change of glycine concentration. It has been reported that gut microbiota can signal to the brain through the regulation of tryptophan metabolism, thus affecting the development, function and behavior of the brain, leading to the occurrence of neurodevelopmental diseases derived from gut microbiota [47]. In our study, the hippurate concentration in ACTH group was significantly higher than that in Control group (Additional file 2: Table S1), and a critical signal molecule in the brain-gut axis, 5-HT, was significantly decreased (Additional file 1: Fig. S2), possibly leading to the occurrence of brain-gut axis disorders and depression.

In addition, ascorbate and aldarate metabolism was also disrupted in ACTH-induced depression rats compared to Control group. Ascorbic acid, an important antioxidant in the CNS, promotes the synthesis of neurotransmitters (5-HT and NE) [48]. Compared with Control group, the level of ascorbic acid in ACTH group was significantly reduced (Additional file 2: Table S1), resulting in lower levels of 5-HT and NE in serum (Additional file 1: Fig. S2). The abnormality of these monoamines is widely accepted as the main biological mechanism of depression, and targeted by most of antidepressants.

Myo-inositol, a stereoisomer of inositol, is a carbocyclic sugar, which is widely present in brain and other mammalian tissues. It mediates cell signal transduction through various hormones, neurotransmitters and growth factors and is involved in osmoregulation. Myo-inositol has the highest concentration in the brain and plays an important role here, combining other neurotransmitters and some steroid hormones with their receptors [49]. It’s also an essential participant and regulator of inositol phosphate metabolism [50]. Chiappelli et al. [51] found that the occurrence of depression in schizophrenic patients was closely related to the reduction of myo-inositol levels by proton magnetic resonance spectroscopy analysis. This finding is consistent with the decrease in myo-inositol levels in ACTH group (Additional file 2: Table S1).

In the microbial community profiling, the PLS-DA analysis and the compositional structure both presented the significant differentiation between the groups (Fig. 4a, b). According to the reports, the increase of the ratio of *Firmicutes* to *Bacteroidetes* of phylum level was commonly observed in both depression patients [52] and patients with irritable bowel syndrome [53]. Therefore, the increased ratio of *Firmicutes* to *Bacteroidetes* is considered to be a marker of the gut–brain axis disorders, which also occurred in ACTH group (Additional file 1: Fig. S4).

The LEfSe difference analysis showed that the differential microbiota was mainly concentrated on the cumulative number of *Oscillospira* and *Ruminococcus* (Fig. 4c). *Oscillospira* is a mysterious bacterial genus that has never been cultured but is continuously detected by sequencing of 16S rRNA gene in the human microbiome [54]. In terms of the metabolites excreted by *Oscillospira*, it is inversely correlated with inflammatory diseases and BMI [55]. BMI represented the best predictive value for gut dysbiosis and metabolic alterations. It is speculated that *Oscillospira* may produce short-chain fatty acid butyrate, similar to trends of other genera (e.g., *Roseburia* and *Faecalibacterium*) [56]. Butyrate concentrations were negatively correlated with anxiety levels. In addition, *Ruminococcus*, a genus of class *Clostridia* which pointing to an energy metabolism dysfunction, was identified as one of characteristic genus of individuals with inflammatory bowel disease (IBD) [57].

By integrating gut microbiota data with differential metabolites, our results highlighted the tight crosstalk between the gut microbiota and host metabolism in ACTH-induced depression rats. As shown in Fig. 5, the relevant genus of gut microbiota predicted from each of differential urinary metabolites were *Ruminococcus*, *Akkermansia*, *Lactobacillus* and *Klebsiella*. *Akkermansia muciniphila*, the only currently known species within genus *Akkermansia*, has the ability to continuity regeneration of the intestinal epithelium and degrade mucin, and also producing acetic acid, propionic acid and oligosaccharides. These products become the substrate for *Faecalibacterium prausnitzii*, one of the main producers of butyrate in the intestine. Anaerobic bacteria that produce butyrate help to inhibit inflammation in the gastrointestinal tract, and prevent an increase in intestinal permeability [58]. In our study, 6 metabolites were statistically relevant to *Akkermansia*, including pyruvic acid, 4-hydroxybenzoic acid, myo-inositol, hippurate, d-arabitol and ascorbic acid, many of which are closely related to depression, as we discussed above.

*Lactobacillus* is a genus of Gram-positive, facultative anaerobic or microaerophilic, is the main component the lactic acid bacteria group (i.e. they convert sugars to lactic acid). It was reported ingestion of *Lactobacillus* strain could regulate emotional behavior and central GABA receptor expression in mouse via the vagus nerve [59], as well as improve behavioral, cognitive, and biochemical aberrations caused by chronic restraint stress [60]. As shown in Fig. 5, there are 3 metabolites relevant to *Lactobacillus*, namely pyruvic acid, hippurate and d-arabitol. Arabitol is a sugar alcohol, which can be formed by reducing arabinose or lyxose. Some organic acid tests examined the presence of d-arabitol, which may indicate overgrowth of intestinal microbes. Arabitol showed very similar inhibition effects to its isomer xylitol on oral *Lactobacilli* [61]. As we discussed above, the correlation analysis between differential metabolites and bacteria revealed that active metabolites of *Akkermansia* and *Lactobacillus* were closely related to the host inositol phosphate metabolism, phenylalanine, tyrosine and tryptophan biosynthesis, which indicated there was a significant crosstalk between the gut microbiota and the host metabolism in ACTH-induced TRD rats.

In addition, it has been reported that the abundance of *Klebsiella* and *Ruminococcus* was changed in patients with MDD compared with healthy controls [62, 63]. In our study, these two intestinal microbes were observed to have varying degrees of correlation with l-threonine, pyruvic acid, mannitol and hippurate, which are closely related to the development of depression as we discussed earlier, but there was no statistical significance (*P* > 0.05). These results suggest that the changes in gut microbiota and urinary metabolic phenotype are associated with the development of depression. The relevance between the gut microbiota of genus level and the differential urinary metabolites further confirmed the correlation between disturbances of gut microbiota and the disturbed microorganism-host metabolic balance in ACTH-treated rats.

## Conclusions

In this study, 16S rRNA gene sequencing was combined with GC-TOFMS based metabolomics to assess effects of ACTH-treatment on gut microbial community profiling and urinary metabolomic signatures. Our results showed that ACTH-induced depression not only disturbs the composition of gut microbiota, such as *Oscillospira*, *Ruminococcus*, *Akkermansia*, *Lactobacillus* and *Klebsiella*, but also alters the homeostasis of metabolic pathway that including pyruvate metabolism, ascorbate and aldarate metabolism, inositol phosphate metabolism, glycine, serine and threonine metabolism, and glycolysis or gluconeogenesis. In addition, the correlation analysis revealed that altered gut microbes were closely related to differential urinary metabolites. These gut microbiota with strong host metabolic connectivity can provide a basis for the disorder of gut microbiota community in ACTH-induced depression.

## Additional files

**Additional file 1: Fig S1.** Neurobehavioral alternations in ACTH-treated rats. (A) Immobile time of forced swimming test (FST). (B) Immobile time of tail suspension test (TST). Data are expressed as mean ± SD (n = 10). **P < 0.01 compared with Control group. **Fig. S2.** Changes of serum biochemical parameters in ACTH-induced depression rats. The serum concentrations of 5-HT (A), NE (B), CRH (C), ACTH (D), and CORT (E). Data are expressed as mean ± SD (n = 10). ***P < 0.001, **P < 0.01 compared with Control group. **Fig. S3.** OPLS-DA score plots showing the distributions of urinary metabolites in ACTH group and Control group (n = 6). **Fig. S4.** Ratio of Firmicutes to Bacteroidetes in ACTH group and Control group. Data are expressed as mean ± SD (n = 6). **P < 0.05 compared with Control group. **Fig. S5.** (A) Genus-level taxonomic distributions of the microbial communities in fecal contents. (B) The relative abundance of bacterial genus detected in fecal samples. Data are expressed as mean ± SD (n = 6). *P < 0.05, **P < 0.01 compared with Control group.

**Additional file 2: Table S1.** Differential metabolites between ACTH group and Control group.

## Data Availability

The data are included in the article as figures and its additional files.

## Abbreviations

ACTH: adrenocorticotrophic hormone
GC-TOFMS: gas chromatography-time-of-flight mass spectrometer
WHO: World Health Organization
TRD: treatment resistant depression
HPA: hypothalamic–pituitary–adrenal
5-HT: 5-hydroxytryptamine
NA: noradrenaline
CRH: corticotropin-releasing hormone
FST: forced swimming test
CNS: central nervous system
NMDAR: *N*-methyl-d-aspartate receptor
TST: tail suspension test
NE: norepinephrine
CORT: cortisol
PCA: principal component analysis
OPLS-DA: orthogonal partial least squares discriminant analysis
VIP: variable importance in the projection
FC: fold change
KEGG: Kyoto Encyclopedia of Genes and Genomes
HMDB: Human Metabolome Database
OTUs: operational taxonomic units
PLS-DA: partial least squares discriminant analysis
LDA: linear discriminant analysis
LEfSe: linear discriminant analysis effect size
SD: standard deviation
SCFAs: short-chain fatty acids
TCA: tricarboxylic acid
IBD: inflammatory bowel disease
