# Supplementary material for: Metabolomic signatures and microbial community profiling of depressive rat model induced by adrenocorticotrophic hormone
Source: J Transl Med. 2019 Jul 15;17:224. doi: 10.1186/s12967-019-1970-8 (PMC6631535; doi:10.1186/s12967-019-1970-8)
Supplement: Supplementary file 1 — Additional file 1: Fig S1. Neurobehavioral alternations in ACTH-treated rats. (A) Immobile time of forced swimming test (FST). (B) Immobile time of tail suspension test (TST). Data are expressed as mean ± SD (n = 10). **P < 0.01 compared with Control group. Fig. S2. Changes of serum biochemical parameters in ACTH-induced depression rats. The serum concentrations of 5-HT (A), NE (B), CRH (C), ACTH (D), and CORT (E). Data are expressed as mean ± SD (n = 10). ***P < 0.001, **P < 0.01 compared with Control group. Fig. S3. OPLS-DA score plots showing the distributions of urinary metabolites in ACTH group and Control group (n = 6). Fig. S4. Ratio of Firmicutes to Bacteroidetes in ACTH group and Control group. Data are expressed as mean ± SD (n = 6). **P < 0.05 compared with Control group. Fig. S5. (A) Genus-level taxonomic distributions of the microbial communities in fecal contents. (B) The relative abundance of bacterial genus detected in fecal samples. Data are expressed as mean ± SD (n = 6). *P < 0.05, **P < 0.01 compared with Control group. [file 12967_2019_1970_MOESM1_ESM.docx]

**
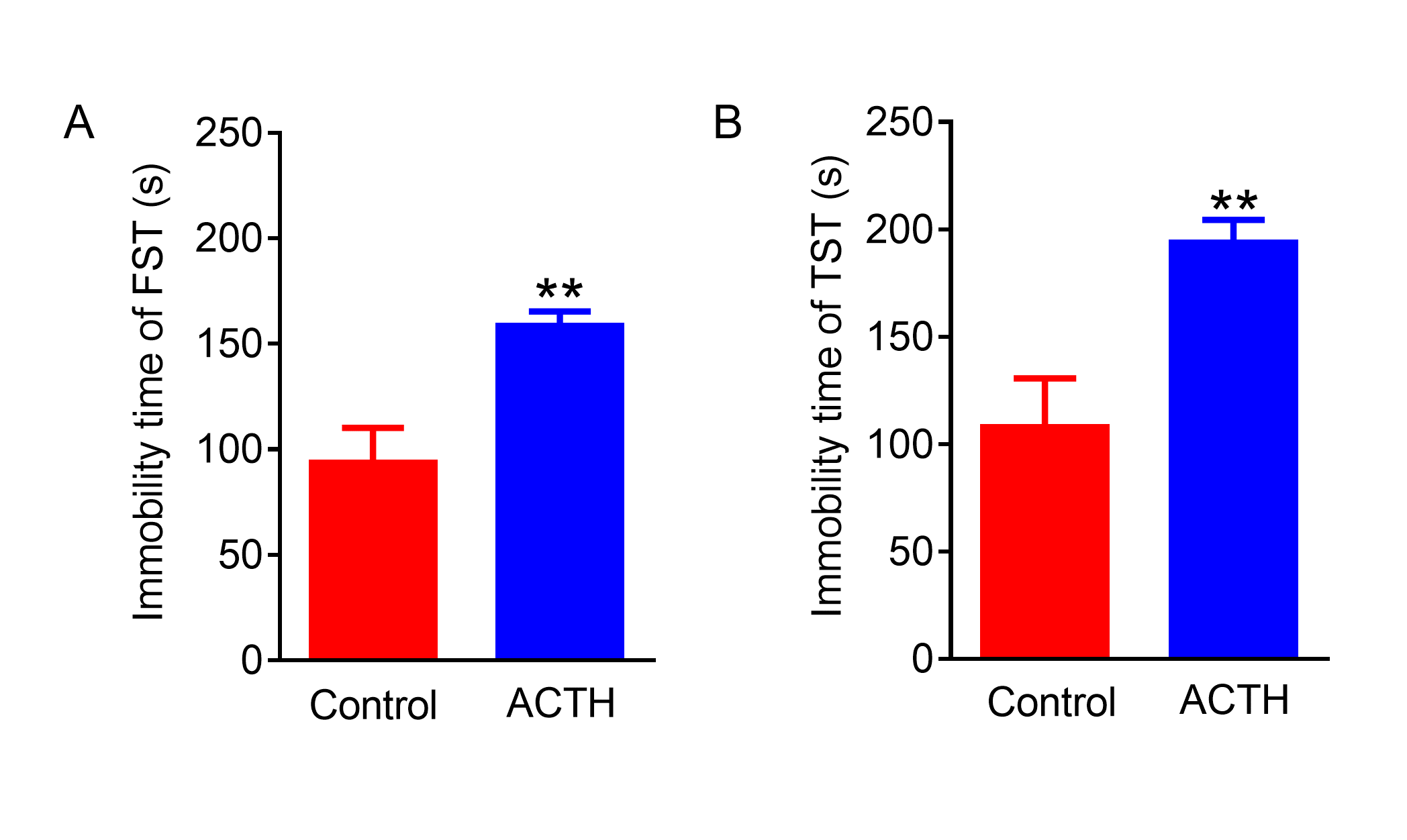
Fig. S1 Neurobehavioral alternations in ACTH-treated rats.** (A) Immobile time of forced swimming test (FST). (B) Immobile time of tail suspension test (TST). Data are expressed as mean ± SD (n = 10). ^**^ *P* <0.01 compared with Control group.

**
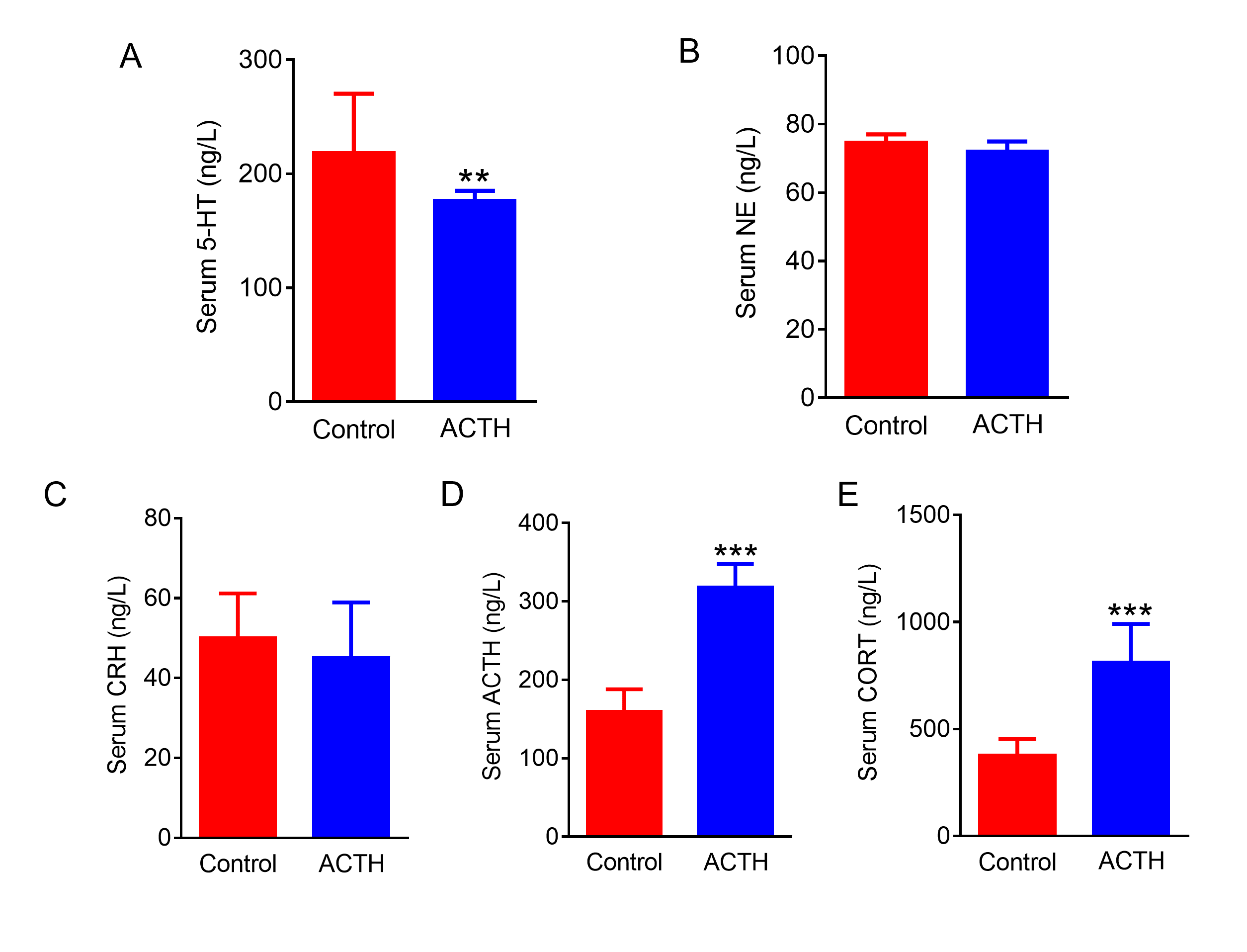
Fig. S2 Changes of serum biochemical parameters in ACTH-induced depression rats.** The serum concentrations of 5-HT (A), NE (B), CRH (C), ACTH (D), and CORT (E). Data are expressed as mean ± SD (n = 10). ^***^ *P* <0.001, ^**^ *P* <0.01 compared with Control group.

**
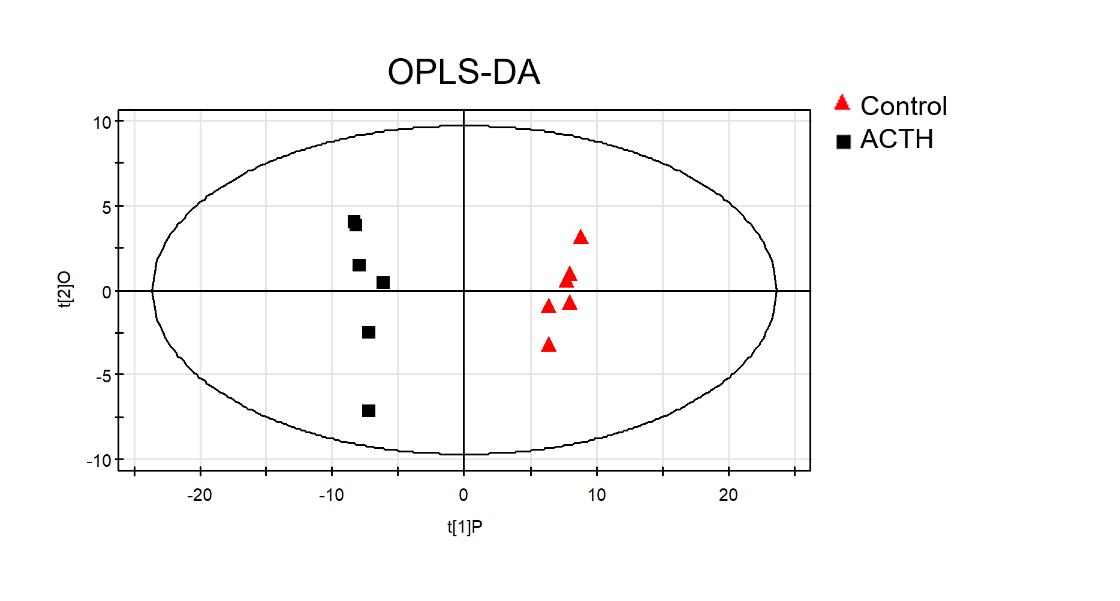
**

**Fig. S3** OPLS-DA score plots showing the distributions of urinary metabolites in ACTH group and Control group (n = 6).

**
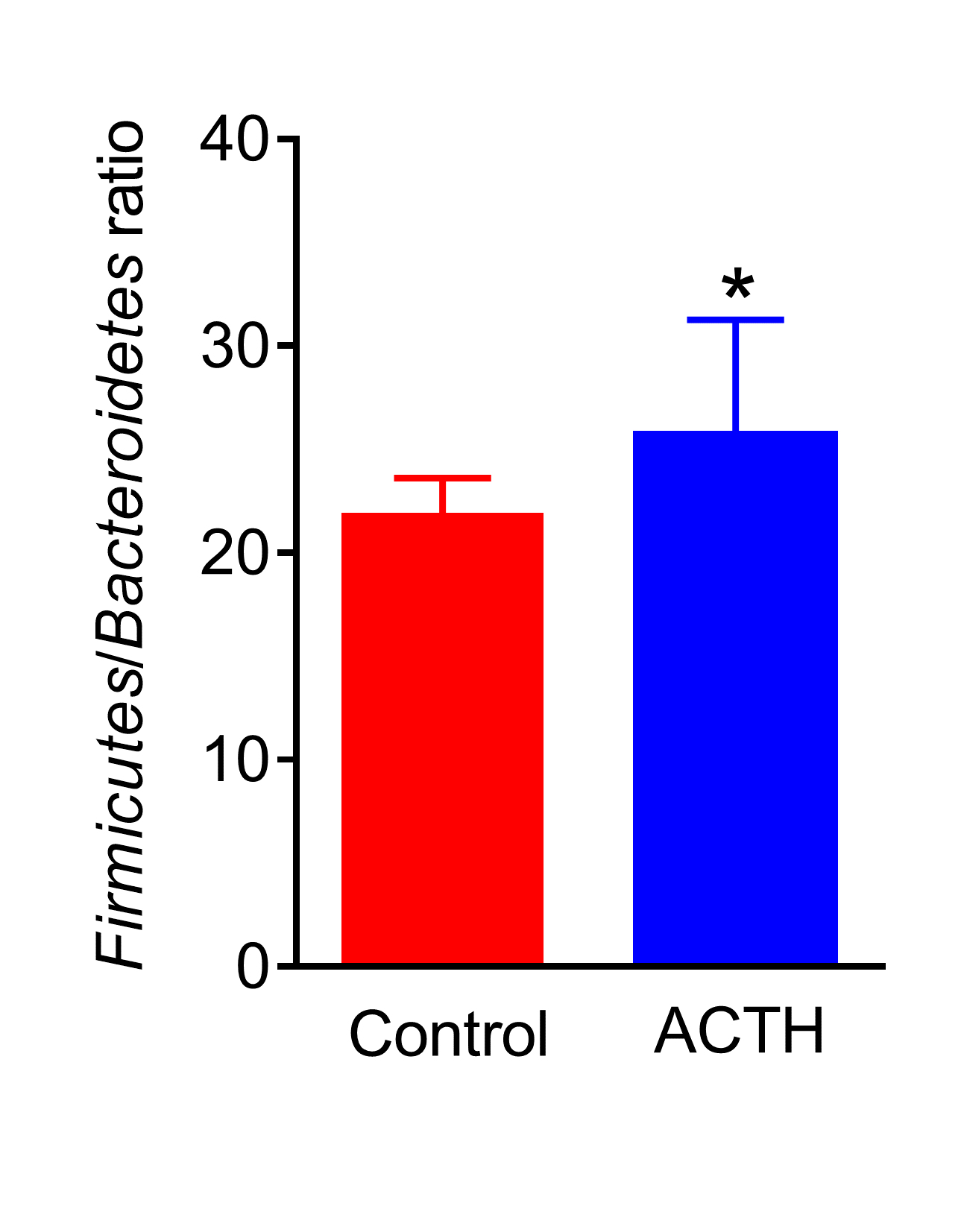
**

**Fig. S4** Ratio of *Firmicutes* to *Bacteroidetes* in ACTH group and Control group. Data are expressed as mean ± SD (n = 6). ^*^*P* < 0.05 compared with Control group.

**
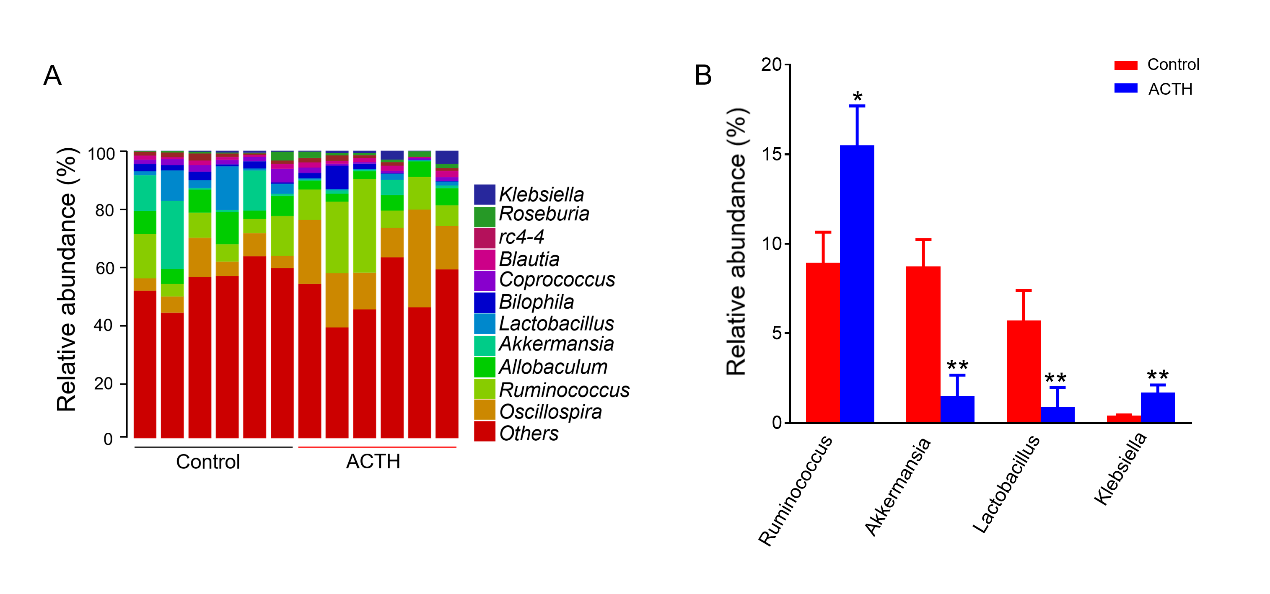
Fig. S5 (A)** Genus-level taxonomic distributions of the microbial communities in fecal contents. **(B)** The relative abundance of bacterial genus detected in fecal samples. Data are expressed as mean ± SD (n = 6). ^*^*P* <0.05, ^**^*P* <0.01 compared with Control group.
