# Supplementary material for: Metabolomic signatures and microbial community profiling of depressive rat model induced by adrenocorticotrophic hormone
Source: J Transl Med. 2019 Jul 15;17:224. doi: 10.1186/s12967-019-1970-8 (PMC6631535; doi:10.1186/s12967-019-1970-8)
Supplement: Supplementary file 2 — Additional file 2: Table S1. Differential metabolites between ACTH group and Control group. [file 12967_2019_1970_MOESM2_ESM.docx]

**Table S1 Differential metabolites between ACTH group and Control group.**

| **Metabolites** | **RT (min)** | **Mass** | **ACTH *vs.* Control** | | | **Metabolic Pathways** |
| --- | --- | --- | --- | --- | --- | --- |
|  |  |  | **Fold Change^a^** | **VIP^b^** | **T-test (*P*)** |  |
| **Pyruvic acid** | 5.23 | 174 | 0.22 | 1.32 | ＜0.001 | Pyruvate metabolism; Glycolysis or gluconeogenesis; Glycine, serine and threonine metabolism; Ascorbate and aldarate metabolism |
| **L-threonine** | 16.82 | 218 | 0.34 | 1.31 | ＜0.001 | Glycine, serine and threonine metabolism |
| **Myo-inositol** | 25.99 | 217 | 0.32 | 1.21 | ＜0.01 | Inositol phosphate metabolism |
| **Ascorbic acid** | 33.59 | 332 | 0.29 | 1.29 | ＜0.01 | Ascorbate and aldarate metabolism |
| **Mannitol** | 26.40 | 319 | 0.35 | 1.27 | ＜0.001 | others |
| **D-gluconic acid** | 24.32 | 217 | 0.30 | 1.24 | ＜0.001 | others |
| **4-hydroxybenzoic acid** | 15.32 | 223 | 0.52 | 1.23 | ＜0.001 | others |
| **D-arabitol** | 17.45 | 217 | 0.32 | 1.30 | ＜0.001 | others |
| **Hippurate** | 20.27 | 105 | 17.74 | 1.26 | ＜0.01 | Phenylalanine, tyrosine and tryptophan biosynthesis |

**a** Fold change was calculated as the ratio of the average relative level between the two groups (FC value = ACTH/Control).

**b** VIP was obtained from OPLS-DA with a threshold of 1.0.
